# Kinetic analysis of the isoleucyl‐tRNA synthetase mechanism: the next reaction cycle can start before the previous one ends

**DOI:** 10.1002/2211-5463.12362

**Published:** 2017-12-20

**Authors:** R. Kalervo Airas

**Affiliations:** ^1^ Department of Biochemistry University of Turku Finland

**Keywords:** isoleucyl‐tRNA synthetase, kinetics, magnesium, tRNA

## Abstract

Aminoacyl‐tRNA synthetases join correct amino acids to their cognate tRNA at the start of the protein synthesis. Through the kinetic analysis, it is possible to estimate how their functional details correspond to the known structural features. Kinetic analysis of the isoleucyl‐tRNA synthetase (IleRS) from *Escherichia coli* was accomplished. Sixteen different steady‐state two‐ligand experiments were statistically analysed simultaneously so that the same rate equations and same rate and dissociation constants applied to all experiments. The so‐called rapid equilibrium segments procedure was used to derive the rate equations. The final best‐fit mechanism included the normal activation and transfer steps, and reorganization of the steps between them and after the transfer step. In addition, the analysis strongly suggested an additional activation step, formation of a new isoleucyl‐AMP before the isoleucyl‐tRNA was freed from the enzyme. The removal of Ile‐tRNA was possible without the formation of Ile‐AMP if both isoleucine and ATP were bound to the E‐Ile‐tRNA complex, but this route covered only 11% of the total formation of Ile‐tRNA. In addition to the Mg^2+^ in MgATP or MgPP_i_, only two tRNA‐bound Mg^2+^ were required to explain the magnesium dependence in the best‐fit mechanism. The first Mg^2+^ could be present in all steps before the second activation and was obligatory in the first reorganizing step and transfer step. The second Mg^2+^ was present only at the transfer step, whereas elsewhere it prevented the reaction, including the activation reactions. Chloride inhibited the IleRS reaction, while 100 mm KCl caused 50% inhibition if the ionic strength was kept constant with K‐acetate. The $K_{m}^{\mathrm{app}}$ (tRNA) value was increased from 0.057 to 1.37 μm when the KCl concentration was increased from 0 to 200 mm. The total rate equation helps to understand the reaction route and how the simultaneous presence of Ile‐tRNA and Ile‐AMP can cause new possibilities to proofreading mechanisms of this enzyme.

**Enzyme:**

Isoleucyl‐tRNA synthetase (EC 6.1.1.5)

**Abbreviations**
aa: aminoacyl
ArgRS: arginyl‐tRNA synthetase
IleRS: isoleucyl‐tRNA synthetase

The basic mechanism of the aminoacyl‐tRNA synthetases includes the formation of aminoacyl‐AMP in the activation reaction and thereafter the transfer of the amino acid from aa‐AMP to tRNA in the transfer reaction. The basic mechanism was elucidated quite early after the discovery of the aminoacyl‐tRNA synthetases [reviews see Refs 1, 2]. Thereafter, new details of the reaction have been the subject of continuous study, including the detailed crystal structure 3. Much attention has been paid to the editing mechanisms, which eliminate the erroneously formed products, pretransfer proofreading at the aa‐AMP level and post‐transfer proofreading at the aa‐tRNA level 4, 5. The aminoacyl‐tRNA synthetases have been divided into two classes according to structural and functional properties 6, 7. Isoleucyl‐tRNA synthetase (IleRS) falls into Class Ia together with other synthetases for the branched chain amino acids.

A decade ago, I published a best‐fit analysis of the arginyl‐tRNA synthetase (ArgRS) reaction 8 where numerous two‐ligand experiments were simultaneously analysed. In this study, the same approach is repeated with the IleRS from *Escherichia coli*. Both IleRS and ArgRS belong to the Class I aminoacyl‐tRNA synthetases and have close structural similarities 9.

In some previous works, I have applied the ‘rapid equilibrium segments’ procedure to derive rate equations for aminoacyl‐tRNA synthetases 10, 11. These analyses included the dependences on the magnesium and polyamine concentrations and the dissociation of Mg^2+^ from Ile‐tRNA before it is freed from the enzyme 10, the differences in the magnesium dependences between the Class I and Class II aminoacyl‐tRNA synthetases 11, and chloride inhibition 12. Due to limited experimental material, those studies did not lead to equations that could be satisfactorily used for new different experiments. In this study, the procedure has been improved by simultaneously analysing 16 different two‐ligand experiments and, in addition, avoiding the inhibiting chloride and sulphate in adjusting the Mg^2+^ concentration. The optimal statistical result required a step at the end of the reaction cycle where Ile‐AMP for the next cycle was formed before the Ile‐tRNA was liberated from the enzyme. This revives the old reaction cycle suggested by Yarus and Berg 13. Apparently, the reaction site is opened when the CCA‐Ile end of tRNA is turned to a separate editing site for the post‐transfer proofreading 3. So the formation of a new Ile‐AMP could be possible on the reaction site before the Ile‐tRNA is totally removed from the enzyme. This, also, increases the number of the enzyme intermediates, which could be involved in the pretransfer proofreading.

## Experimental procedures

### Materials

Isoleucyl‐tRNA synthetase was purified from *E. coli* B as described previously 14. Unfractionated tRNA from *E. coli* MRE 600 (Boehringer) was used.

### Enzyme assays

The rate of the aminoacylation of tRNA was assayed with the filter paper–acid precipitation method 15 with modifications. The reaction mixture (100 μL) contained 50 mm Hepes/25 mm KOH (pH 7.4 at 30 °C), 0.02% chicken egg albumin, 1 mg·mL^−1^ of tRNA (1.1 μm tRNA^Ile^), 2 mm ATP, 5 μm nonradioactive Ile, about 60 000 cpm of [^14^C]Ile (0.9 μm), 3 mm Mg‐(acetate)_2_ (1 mm excess Mg^2+^), 50 mm K‐acetate, 1 mm dithiothreitol and the enzyme. The reaction temperature was 30 °C. 180 μm Mg^2+^ was carried to the reaction mixture by tRNA, and was taken into account in the calculations. The reaction was stopped by pipetting a sample (40 μL) onto Whatman 3MM paper placed close to the surface of 50% formic acid, which reduces the pH below 4 in seconds. The paper pieces were washed three times with a solution containing 0.2 m HCl and 5% acetic acid and finally once with ethanol. The paper pieces were counted for radioactivity of the formed aminoacyl‐tRNA. The initial rates were calculated using the integrated Michaelis equation containing the product inhibition 16 to avoid the slight curvature in the rate curves caused by the formed Ile‐tRNA.

The ATP‐PP_i_ exchange activities were measured in a similar reaction mixture to the aminoacylation, but ^32^PP_i_ (50 000–300 000 cpm, 0.05–1 μm) was substituted for the radioactive amino acid and 50 μm nonradioactive PP_i_ was added. The Ile concentration was 50 μm. The product radioactive ATP was separated from the radioactive PP_i_ by paper chromatography as described previously 14.

### Equations and best‐fit analysis

The equations were derived as described previously 8, 10. The following steps were included:

1. The total reaction scheme was divided into 4–6 segments, depending on the mechanism to be tested. The enzyme intermediates within a segment should be in equilibrium with each other. The *C* and *D* terms were defined to express the total enzyme intermediate concentrations in the segment (*D*_i_*[E_*i*_]) and the rates between the segments (*C*_ij_*[E_*i*_]).
2. The steady‐state rate equations were derived for the ATP‐PP_i_ exchange reaction and the aminoacylation of tRNA using the *C* and *D* terms. The segments were handled like the enzyme intermediates in the normal derivation of rate equations.
3. In the rate equation algorithm, the *C* and *D* terms were expressed using the real rate and equilibrium constants and ligand concentrations.
4. The algorithm and the rate equations (from step 2) were used to obtain the calculated rate values. These calculated rate values and the measured rate values were used in the best‐fit analysis.

The best‐fit analysis was performed using the nonlinear regression with the least squares of the residuals between the measured rate and calculated rate values. The kinetic constants were refined by successive iterations. The variances and standard errors were calculated as described 8. The sum of the variances of the different experiments was minimized. The minimization was performed by systematically changing (in 5%, 1% or 0.2% steps) the value of a kinetic constant in the rate equation algorithm, choosing the value giving the lowest sum of variances and doing the same with all (39) constants one by one. As the values of the kinetic constants are not independent of each other, the procedure is repeated so many times that the sum of the variances and the values of the constants do not change. The order of the constants in the row should be varied, too.

## Results

### Measurements

**Figure 1:**
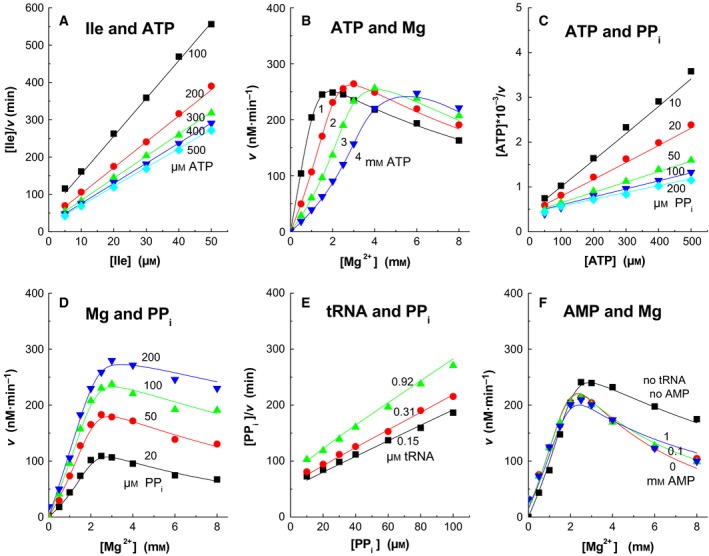
ATP‐PP_i_ exchange rates at different conditions. The lines were calculated using the rate equation algorithm, Eqns (2) or (3), and the optimized constant values from Table 1. The total Mg^2+^ ion concentrations were in (A) [MgATP] + 1 mm; (C) [MgATP] + [MgPP_i_] + 1 mm; D, [MgPP_i_] + [Mg‐acetate]; E, [MgPP_i_] + 4 mm; and in (B) and (F) as shown. tRNA was not present in (A), (B), (C) and (D). The standard errors were (A) 2.1%; (B) 3.2%; (C) 4.2%; (D) 4.7%; (E) 3.0%; and (F) 7.4%.

**Figure 2:**
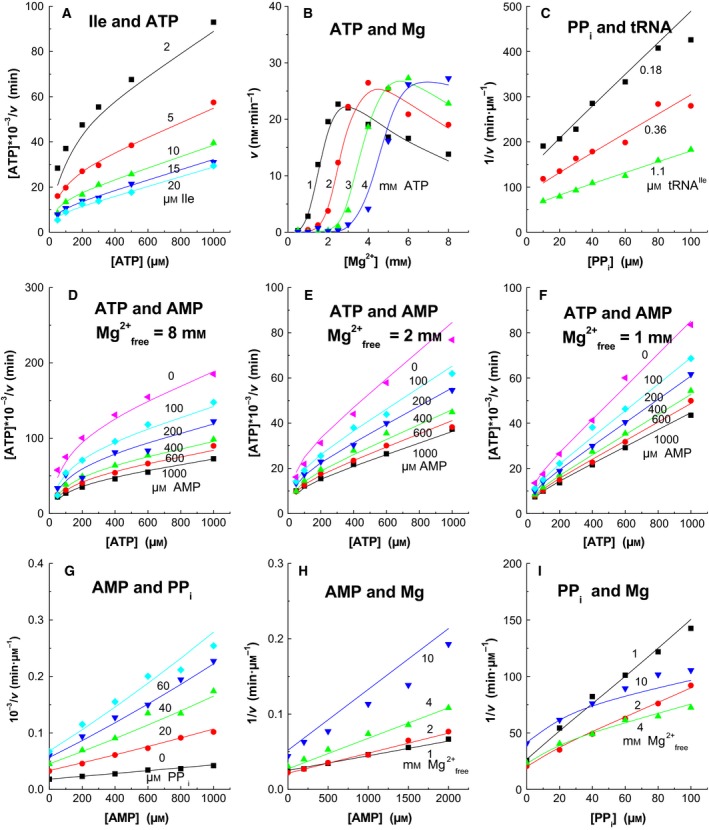
Aminoacylation of tRNA at different conditions. Equation (1) was used in the calculations of the lines. The total Mg^2+^ ion concentrations were in (A) [MgATP] + 4 mm; (B) as shown; (C) [MgPP_i_] + 4 mm; (D) [MgATP] + 8 mm; (E) [MgATP] + 2 mm; (F) [MgATP] + 1 mm; (G) 4 mm; (H) [Mg‐acetate] + 1 mm; (I) [MgATP] + [Mg‐acetate]. The standard errors were in (A) 5.0%; (B) 9.8%; (C) 5.3%; (D) 6.7%; (E) 5.1%; (F) 3.7%; (G) 3.7%; (H) 6.9%; and (I) 6.1%.

The kinetic measurements for the statistical analysis are presented in Figs 1 and 2. They all are two‐ligand steady‐state experiments where either the ATP‐PP_i_ exchange or the aminoacylation of tRNA is measured. The experiments were chosen to cover the different parts of the total reaction. For instance, the changes in the ATP and isoleucine concentrations (in segment 1) or PP_i_ concentrations (segment 2) or AMP concentrations (segment 3) describe mainly the events close to the segments where these ligands are bound. In the aminoacylation reactions (Fig. 2), the products PP_i_ and AMP inhibit, but in the ATP‐PP_i_ exchange reaction PP_i_ works like substrate. The simultaneous analysis of all experiments requires that they have been performed under similar conditions. The potassium concentration used here was 75 mm, which resulted from 50 mm K‐acetate and the buffer. Chloride and sulfate were avoided due to their inhibiting effect. Their concentrations are low in *E. coli* cells 17, 18. Mg‐acetate was used instead of MgCl_2_ or MgSO_4_ to adjust the magnesium concentration. The substrate concentration dependences were plotted as Hanes plots (*s*/*v* vs. *s*) and the inhibition dependences as Dixon plots (1/*v* vs. *i*). Due to the complexity of the reaction, the Hanes and Dixon plots are seldom straight lines and the *v* vs. [Mg^2+^] plots are not hyperbolas.

**Figure 3:**
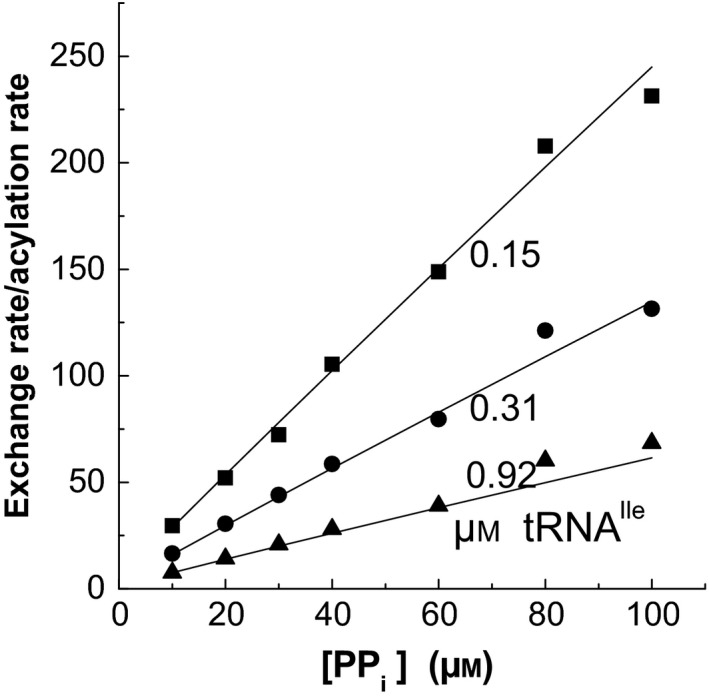
Relation of the rates of ATP‐PP_i_ exchange and aminoacylation of tRNA. The lines were calculated using equations (1) and (2) and the optimized constant values. The exchange and aminoacylation rates were measured at identical conditions, only the radioisotope was changed. ATP was 2 mm, Ile 10 μm and total Mg^2+^ 4 mm + [MgPP_i_]. The standard error was 7.0%.

In Fig. 3, the relation between the ATP‐PP_i_ exchange and aminoacylation rates, *v*_exch_/*v*_acyl_, was determined, and these results were used in the statistical analysis. This relation gives a simple expression if the second activation reaction does not exist 8, but the second activation makes the relation more complicated.

### Best‐fit model

In the best‐fit analysis, the sum of the squares of the residuals between the measured rate and calculated rate values was used to calculate the error percentages for all two‐ligand experiments in Figs 1, 2, 3. The sum of the error percentages was minimized.

**Figure 4:**
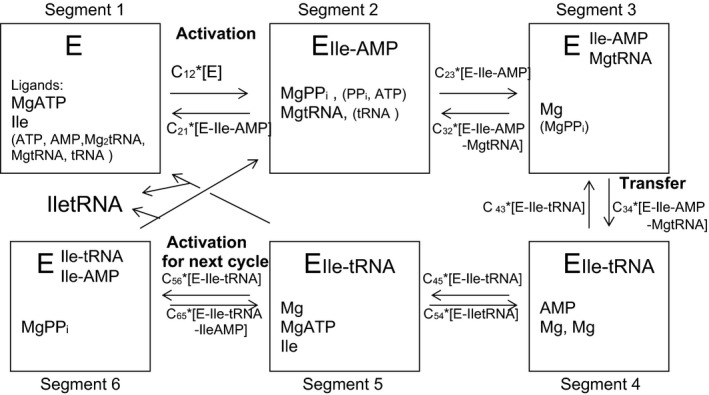
Division of the isoleucyl‐tRNA synthetase reaction in six segments. The central enzyme intermediate and the ligands, which are bound to it in each segment, are indicated. The sum of the enzyme intermediate concentration in the segment *i* is *D*_i_*[E_*i*_], where E_*i*_ is the central intermediate. The rate from segment *i* to segment *j* is *C*_ij_*[E_*i*_]. The expressions of the *D*_*i*_ and *C*_*ij*_ terms are written in the rate equation algorithm.

The scheme of the best‐fit model of the reaction is given in Fig. 4. The reaction is divided into six segments according to the procedure of the ‘rapid equilibrium segments’ 19. The reaction scheme follows mainly the known reaction mechanism 1, 2, 3, 4, 5 until segment 4, including the activation and transfer reactions and the binding modes of the substrates. The role of the separate post‐transfer editing site, which as well is a known structure 3, 5, becomes evident in segments 5–6. It gives the possibility for the CCA‐Ile end of tRNA and for Ile‐AMP to be bound to different sites. Although there seems not to be any discrepancy between this structure and the present kinetic model, the kinetic details remain to be adjusted.

The total rate is slow (*k*_cat_ = 0.7 s^−1^) allowing the equilibrium to be settled in the different segments. The reaction can run either through segment 5 → segment 1 or through segment 5 → segment 6 → segment 2. The rate equation is an algorithm which is shown in the scheme shown in Fig. 4. The rate of the aminoacylation of tRNA is shown in Eqn (1). The rate of the ATP‐PP_i_ exchange in the presence of tRNA and in the absence of tRNA is in Eqn (2) and in Eqn (3), respectively. The values of the kinetic constants in the best‐fit system are in Table 1. If a ligand exists similarly in a segment and the reactions from and to the segment, the terms containing the ligand need not to be written in the equations. Therefore, tRNA has not been written in *D*_3_ and Ile‐tRNA is not in *D*_4_, *D*_5_ or *D*_6_.

**Table 1:** The best‐fit values of the kinetic constants of the IleRS reaction. The *k*_cat_ value at optimal reaction conditions was 0.71 s^−1^

Constant	Definition	Unit	Value	Range
*K*_1M_	MgATP·E	μm	371	300–440
*K*_1_	free ATP·E	μm	1043	500–
*K*_AM_	MgATP·E(Ile‐tRNA)	μm	2077	1820–2400
*K*_A2_	Free ATP·E(Ile‐AMP)	μm	746	610–940
*K*_2_	Ile·E	μm	6.63	5.8–7.3
*K*_25_	Ile·E seg. 5	μm	9.28	8.1–10.5
*k*_+3_	Activation	s^−1^	37.7	35.1–41.1
*k*_‐3_	Pyrophosphorolysis	s^−1^	326	313–338
*k*_+35_	Activation, seg. 5	s^−1^	62.8	54–72
*k*_‐35_	Rev. activation, seg. 5	s^−1^	326	210–420
*K*_4M_	MgPP·E	μm	339	327–353
*K*_4_	Free PP_i_·E	μm	46.6	29–97
*K*_4M6_	MgPP·E(Ile‐tRNA)	μm	258	< 930
*K*_5M1_	Mg_2_tRNA·E, seg. 1	μm	0.075	0.065–0.091
*K*_51_	MgtRNA·E, seg. 1	μm	0.206	0.17–0.25
*K*_5F1_	Free tRNA·E, seg. 1	μm	1.7	
*K*_52_	MgtRNA·E, seg. 2	μm	0.297	0.282–0.312
*K*_5F2_	Free tRNA·E, seg. 2	μm	4.7	
*k*_+6C_	Conf. ch.	s^−1^	6.81	6.55–7.09
*k*_‐6C_	Conf. ch.	s^−1^	81.1	77–85
*k*_+6_	Transfer	s^−1^	32.2	30.6–33.8
*k*_‐6_	Reverse transfer	s^−1^	299	272–327
*K*_71_	AMP·E, seg. 1	μm	705	360 <
*K*_74_	AMP·E seg. 4	μm	2198	2020–2410
*k*_+8C_	Conf. ch.	s^−1^	8.39	7.7–9.3
*k*_‐8C_	Conf. ch.	s^−1^	0.436	0.34–0.54
*k*_+8_	Ile‐tRNA·E, seg. 5	s^−1^	0.048	< 0.29
*k*_+8ATP_	Ile‐tRNA·E(ATP), seg. 5	s^−1^	0.02	< 4.1
*k*_+8ILE_	Ile‐tRNA·E(Ile), seg. 5	s^−1^	0.466	0.11–0.82
*k*_+8M_	Ile‐tRNA·E(Mg), seg. 5	s^−1^	0.022	0.014–0.03
*k*_+8SA_	Ile‐tRNA·E(Ile)(ATP), seg. 5	s^−1^	7.0	< 15.7
*k*_+86_	Ile‐tRNA·E(Ile‐AMP), seg. 6	s^−1^	4.15	2.5–10.3
*k*_+86P_	Ile‐tRNA·E(Ile‐AMP)(MgPP)	s^−1^	262	170–440
*K*_MR_	Mg·tRNA	μm	2489	2160–2820
*K*_MR2_	MgtRNA·Mg	μm	1635	1480–1830
*K*_ME3_	Mg·E(MgtRNA)(Ile‐AMP)	μm	2580	2460–2710
*K*_ME4_	Mg·E(Ile‐tRNA)	μm	1515	1200–1890
*K*_ME42_	Mg·E(Ile‐tRNA)(Mg)	μm	6095	5570–6720
*K*_ME5_	Mg·E(Ile‐tRNA)	μm	117	103–132

If all rate constants are multiplied by the same number, the error percentages and thus the whole best‐fit system remain the same, and the dissociation constants remain unchanged. The level of the rate constants was chosen to give the *k*_cat_ value measured at optimal conditions, 0.7 s^−1^. To find the main route of the product dissociation, the removal of the product Ile‐tRNA from the enzyme complex is divided into seven different rate constants *k*_8X_ depending on the enzyme intermediate from which the product is removed.

### Rate equation algorithm

In the algorithm, ATP_f_ means ATP without Mg, and tRNA_f_ is tRNA without Mg.

[tRNA_f_] = [tRNA_tot_]/(1 + ([Mg^2+^]/*K*_MR_)*(1 + [Mg^2+^]/*K*_MR2_))
[MgtRNA] = [tRNA_f_]*([Mg^2+^]/*K*_MR_)
[Mg_2_tRNA] = [tRNA_f_]*([Mg^2+^]/*K*_MR_)*([Mg^2+^]/*K*_MR2_)
*C*_12_ = *k*_3_*(1 + [tRNA_f_]/*K*_5F1_ + [MgtRNA]/*K*_51_)*[Ile]/*K*_2_*[MgATP]/*K*_1M_
*C*_21_ = *k*_‐3_*(1 + [tRNA_f_]/*K*_5F2_ + [MgtRNA]/*K*_52_)*([MgPP_i_]/*K*_4M_)
*C*_23_ = *k*_6C_*[MgtRNA]/*K*_52_*(1 + [MgPPi]/*K*_4M_)
*C*_32_ = *k*_‐6C_*(1 + [MgPPi]/*K*_4M_)
*C*_34_ = *k*_6_*[Mg^2+^]/*K*_ME3_
*C*_43_ = *k*_‐6_*[AMP]/*K*_74_*([Mg^2+^]/*K*_ME4_)*([Mg^2+^]/*K*_ME42_)
*C*_45_ = *k*_8C_*(1 + [Mg^2+^]/*K*_ME4_)
*C*_54_ = *k*_‐8C_*(1 + [Mg^2+^]/*K*_ME5_)
*C*_56_ = *k*_35_*[Ile]/*K*_25_*[MgATP]/*K*_AM_
*C*_65_ = *k*_‐35_*[MgPPi]/*K*_4M6_
*C*_51_ = *k*_8_*(1 + [MgATP]/*K*_AM_+[Ile]/*K*_25_) + *k*_8SA_*([Ile]/*K*_25_)*([MgATP]/*K*_AM_) + *k*_8M_*[Mg^2+^]/*K*_ME5_*(1 + MgATP]/*K*_AM_)
*C*_62_ = *k*_86_ + *k*_86P_*[MgPP_i_]/*K*_4M6_
*D*_1_ = ([Mg_2_tRNA]/*K*_5M1_ + (1 + [tRNA_f_]/*K*_5F1_+[MgtRNA]/*K*_51_)*(1 + [Ile]/*K*_*2*_ *)*)*(1 + [MgATP]/*K*_1M_ + [ATP_f_]/*K*_1_ + [AMP]/*K*_71_)
*D*_2_ = (1 + [MgPP_i_]/*K*_4M_+[PP_i_]/*K*_4_ + [ATP_f_]/*K*_A2_)*(1 + [tRNA_f_]/*K*_5F2_+[MgtRNA]/*K*_52_)
*D*_3_ = (1 + [Mg^2+^]/*K*_ME3_ + [MgPP_i_]/*K*_4M_)
*D*_4_ = (1 + [AMP]/*K*_74_)*(1 + [Mg^2+^]/*K*_ME4_)*(1 + [Mg^2+^]/*K*_ME42_)
*D*_5_ = (1 + [Ile]/*K*_25_+[Mg^2+^]/*K*_ME5_)*(1 + [MgATP]/*K*_AM_)
*D*_6_ = (1 + [MgPP_i_]/*K*_4M6_)
*PAR5* = *C*_65_/*C*_56_ *+C*_62_/*C*_56_
*PAR4* = (*C*_54_/*C*_45_ *+C*_51_/*C*_45_)*PAR5 + C_62_/*C*_45_
*PAR3* = *C*_43_/*C*_34_ *PAR4 + C_51_/*C*_34_ *PAR5 + C_62_/*C*_34_
*PAR2* = *C*_32_/*C*_23_ *PAR3 + C_51_/*C*_23_ *PAR5 + C_62_/*C*_23_
*PAR1* = *C*_21_/*C*_12_ *PAR2 + C_51_/*C*_12_ *PAR5
DENOM = *D*_1_ *PAR1 + D_2_ *PAR2 + D_3_ *PAR3 + D_4_ *PAR4 + D_5_ *PAR5 + D_6_

(1) $$v_{\mathrm{acyl}}=\left(C_{51}*PAR5+C_{62}\right)/\text{DENOM}$$

(2) $$v_{\mathrm{exch}}=\left(C_{21}*PAR2+C_{65}\right)/\text{DENOM}$$

(3) $$v_{\mathrm{exch}}=C_{21}/\left(D_{1}*C_{21}/C_{12}+D_{2}\right)$$

### Description of the details of the reaction in different segments

Segment 1: The substrates isoleucine, MgATP and MgtRNA are written in *D*_1_ to be bound in random order. Mg_2_tRNA is bound but does not stay bound further in *C*_12_ and thus inhibits. Free ATP and AMP compete with MgATP. The binding of the free tRNA (without Mg^2+^) is weak. The reaction *C*_12_ runs both in the presence and in the absence of MgtRNA.

Segment 2: In the best‐fit system, PP_i_ and tRNA can be bound simultaneously, or they do not compete. MgPP_i_ is the reacting form. Mg_2_PP_i_ is not bound, and its dissociation constant becomes high in the statistical analysis. Mg_2_tRNA is not bound, either. In *D*_2_, the free ATP is written to compete with PP_i_. It improves the fit, especially in the ATP‐PP_i_ exchange experiments. MgATP does not have the same effect. The binding of ATP to the PP_i_ site exists in the formation of diadenosine tetraphosphate (Ap_4_A) 20, but this is not further tested here. In the next *C*_23_ step, MgtRNA must be present. MgPP_i_ is not necessary, but does not inhibit the step.

Segment 3: One Mg^2+^ ion is coming to segment 3 in MgtRNA. Another Mg^2+^ ion is bound to the complex at this segment. The next step, the transfer reaction, requires two bound Mg^2+^ ions. MgPP_i_ is not necessary in this segment but it does not inhibit either. In the next *C*_34_ reaction, MgPP_i_ is not present.

Segment 4: AMP and one Mg^2+^ ion must dissociate from the E‐Ile‐tRNA complex in segment 4. The other Mg^2+^ ion can also dissociate, but can as well be present in the further *C*_45_ reaction.

Segment 5: The best‐fit mechanism requires strong binding of Mg^2+^ in segment 5. This can be the same Mg^2+^ ion as before in *C*_45_, but the binding is about 13 times stronger. The Mg^2+^ ion in segment 5 competes with isoleucine (not necessarily for the same binding site), and the presence of Mg^2+^ almost totally prevents the further reactions *C*_51_ and *C*_56_. The further reactions require that both isoleucine and ATP are bound. That is, naturally, obligatory on the route segment 5 → segment 6 → segment 2, but even on the route segment 5 → segment 1 as much as 93% of the reaction goes through the intermediate Ile‐tRNA‐E(Ile)(ATP).

Step *C*_56_ is another activation reaction (like step *C*_12_) for the next reaction cycle. The position of the post‐transfer proofreading should be at segment 5, and therefore, *D*_5_ in fact is more complicated. Apparently, the synthetic site is opened for new Ile and ATP when the CCA‐Ile end of the tRNA turns to the editing site. The removal of Mg^2+^(*K*_ME5_) may have a role in the opening.

Segment 6: Both Ile‐tRNA and Ile‐AMP stay attached to the enzyme. The best‐fit analysis suggests a random dissociation order of PP_i_ and Ile‐tRNA from the enzyme at step *C*_62_.

The calculated amounts of the enzyme intermediates in different segments show that 84% of the enzyme is in segment 2 and 6.6% in segment 6 in the absence of PP_i_ and AMP.

### Chloride inhibition

**Figure 5:**
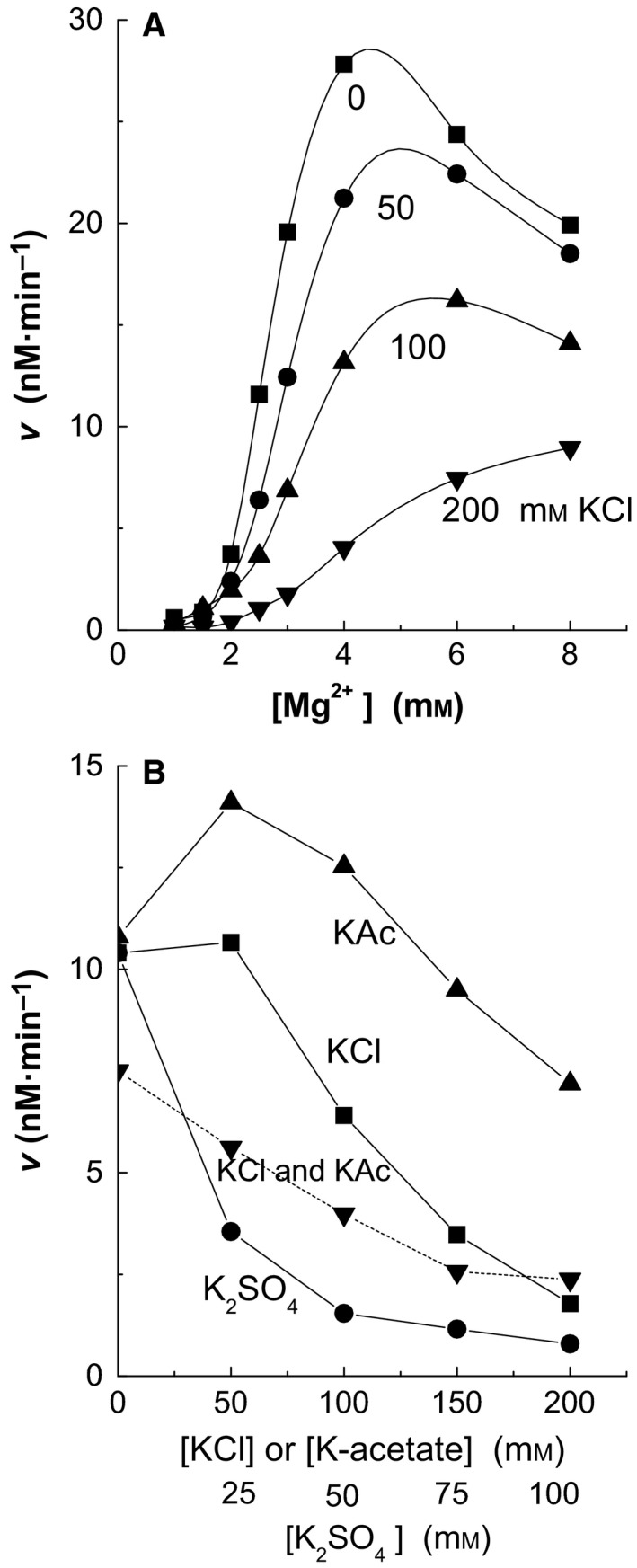
Chloride effect on the aminoacylation reaction. In (A) 50 mm K‐acetate was present in the reaction mixture but in B only when indicated. In the dashed line, the KCl concentration is indicated and K^+^ ion concentration is kept at 200 mm with K‐acetate. ATP was 2 mm, Ile 5 μm, and in (B) Mg‐acetate was 3 mm.

Chloride inhibits the IleRS reaction (Fig. 5). The inhibition is prominent at about a 1 mm concentration of free Mg^2+^ (i.e. 3 mm in Fig. 5A, $Mg_{\mathrm{free}}^{2+}$ + MgATP = 3 mm), which is the concentration of free Mg^2+^ in the cell 21, 22, 23. High ${\text{Mg}}_{\mathrm{free}}^{2+}$ concentrations (> 10 mm) almost eliminate the chloride inhibition. K‐acetate inhibits weakly but K_2_SO_4_ is stronger than KCl (Fig. 5B). KCl and K‐acetate inhibit above 50 mm concentrations when high enough K^+^ concentration for full activity has been reached. If the K^+^ concentration is kept constant at 200 mm, the inhibitory effect of KCl starts from low concentrations; 50 mm K‐acetate was used throughout this study to keep the K^+^ amount high enough (75 mm together with the buffer).

KCl affects the *K*_m_ (tRNA) value. The *K*_m_ values were 0.057, 0.129, 0.334, 0.657 and 1.37 μm at KCl concentrations of 0, 50, 100, 150 and 200 mm, respectively.

The best‐fit analysis for chloride effects was performed using three types of experiments: the *K*_m_ (tRNA) assay, and the experiments similar to Fig. 2H and I at 50 and 150 mm KCl. 150 mm KCl caused an approximately eight times increase in the value of *K*_MR2_ and a twofold increase in *K*_ME42_. The value of *K*_52_ was 0.3, 0.5 or 2.0 μm at 0, 50 and 150 mm KCl, respectively.

The chloride inhibition in IleRS is somewhat stronger than in the arginyl‐tRNA synthetase 8 and tyrosyl‐tRNA synthetase 12. Essentially similar chloride inhibition was observed in the Class II synthetases for phenylalanine, serine, histidine and lysine (not shown) and seems to be a general feature of the aminoacyl‐tRNA synthetases, at least in *E. coli*. The chloride concentration in growing *E. coli* is low (below 25 mm 18 or ‘virtually absent’ 17). Chloride inhibition might make a connection between the rate of protein synthesis and the function of chloride channels and cell potentials 24, 25.

### Effect of spermidine

**Figure 6:**
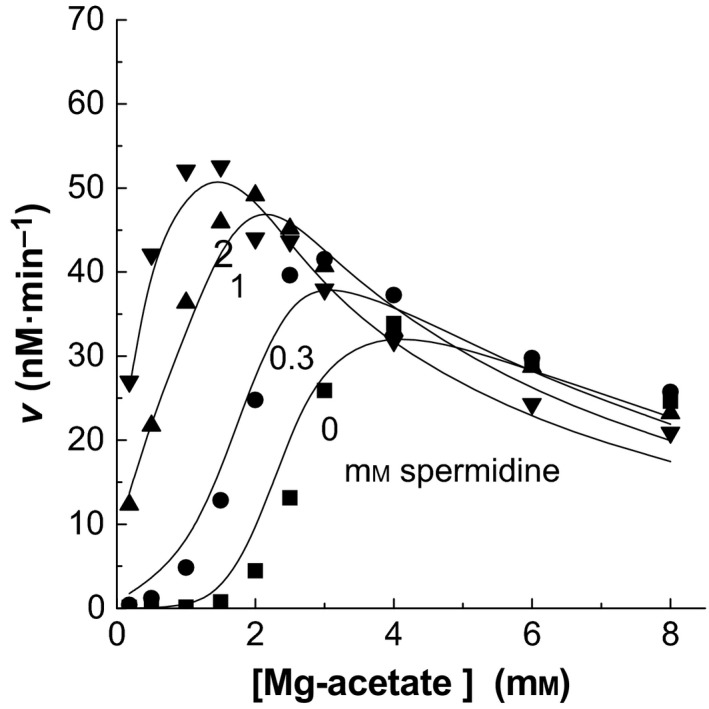
Spermidine effect on the aminoacylation of tRNA. The curves were plotted using the values of the constants from Table 1 and setting [Spd]/*K*_SE3_ to compete with [Mg^2+^]/*K*_ME3_ and [Spd]/*K*_SE42_ to compete with [Mg^2+^]/*K*_ME42_.

Polyamines can replace part of the Mg^2+^ ions in the aminoacyl‐tRNA synthetase reactions 11, 26, 27. Polyamines were not included in the above experiments for the statistical analysis. Only one experiment with spermidine is presented here (Fig. 6). Analysis of spermidine was carried out using the above mechanism and the constant values from Table 1, but spermidine was set to compete with the Mg^2+^ ions in the equations. Only two competition sites were important. Spermidine could replace Mg^2+^ (*K*_ME3_) and Mg^2+^ (*K*_ME42_), which are involved in the forward and backward transfer reaction. The corresponding dissociation constant values were *K*_SE3_ = 470 μm and *K*_SE42_ = 390 μm. The competition with Mg^2+^ (*K*_MR_) was much weaker, *K*_SR_ = 2800 μm. At the other binding sites, no competition was observed.

### Other reaction mechanisms

The above reaction mechanism is the best found so far. If the sum of the error percentages is used as a figure of merit (FOM), it gives the lowest reading, 83.9 per cent units. Some changes can be made in segment 5 without essential weakening the FOM value. If isoleucine and Mg^2+^ (*K*_ME5_) do not compete, the FOM is 86.6. If [Mg^2+^]/*K*_ME5_ is removed from *C*_54_, the FOM is 86.8. In that case, Mg^2+^ (*K*_ME5_) is not on the same binding site as Mg^2+^ (*K*_ME42_), and the backward rate constant *k*_‐8C_ becomes higher, about as high as *k*_+8C_. When Mg^2+^(*K*_ME5_) is removed from segment 5, the FOM rises to 108.

When segment 6 is removed, or the reaction runs through segment 5 → segment 1, the FOM is 90, but even then 99% of the reaction runs through the Ile‐tRNA‐E(ATP)(Ile) complex where both ATP and Ile are bound. If ATP is then removed from segment 5, the FOM is 228; if Ile is removed, it is 128; and if Mg^2+^ (*K*_ME5_) is removed, it is 145.

In all the above cases, the optimization of all constants was carried out, not only the constants involved in the mentioned change.

## Discussion

### Reaction cycle with the formation of second aa‐AMP before dissociation of the product aa‐tRNA

In one of the oldest kinetic works on IleRS, Yarus and Berg 13 studied the binding of tRNA, Ile‐tRNA and modified tRNA:s to the enzyme, and were led to suggest a sequence where the Ile‐AMP for the next reaction cycle was formed before the Ile‐tRNA was freed from the enzyme complex. Eldred and Schimmel 28 also deduced the same reaction cycle. In a thorough kinetic analysis by Freist *et al*. 29, a similar model was one of the possible reaction cycles. Generally, however, such an early formation of Ile‐AMP has not been included in the proposed reaction mechanisms.

An analogous case was found with ArgRS, which normally requires the presence of tRNA for the ATP‐PP_i_ exchange, but the exchange reaction was continued after cessation of tRNA, in the presence of Arg‐tRNA 8.

Although IleRS is an one‐subunit enzyme and has one reaction site, it has an additional editing site for the elimination of wrong amino acids 3, 5. The CCA‐Ile end of the Ile‐tRNA is turned from the reaction site to the editing site. It looks as if the reaction site could be opened for a new Ile‐AMP when the CCA‐Ile end is on the editing site.

The present work suggests that Ile‐tRNA is freed from the enzyme either without the formation of Ile‐AMP in reaction *C*_51_, or after its formation in *C*_62_. The calculated rates show that 89% of the Ile‐tRNA comes through the route segment 5 → segment 6 → segment 2 and only 11% through segment 5 → segment 1 (at 2 mm MgATP, 50 μm Ile and 1 mm free Mg^2+^). Moderate changes in the ligand concentrations do not much change this relation. Only PP_i_ lowers the relation; at 50 μm PP_i_, it is 80%.

### Number of participating Mg^2+^ ions

ATP and PP_i_ always exist in the aminoacyl‐tRNA synthetase reactions as MgATP and MgPP_i_, but Mg_2_PP_i_ seems not to have importance in the IleRS reaction.

Generally, several Mg^2+^ ions are bound to tRNA 30, 31, 32, 33 and are involved in its correct folding 34, 35, 36. However, only few of the tRNA‐bound Mg^2+^ ions are kinetically important 11. Only two tRNA‐bound Mg^2+^ ions are included in the present analysis of IleRS, and one or two Mg^2+^ ions are attached at the same time to the E‐tRNA complex in different steps of the reaction. The equations contain six Mg^2+^ ions (in addition to those in MgATP or MgPP_i_), but some of them must have common binding sites. The terms [Mg^2+^]/*K*_ME3_ and [Mg^2+^]/*K*_ME42_ must refer to the same binding site as both participate only in the transfer reaction, one forward and the other backward, and in addition, spermidine can replace both of these Mg^2+^ ions. This binding not only promotes the transfer reaction but also prevents the other steps, including the activation reactions. According to the equation, the ion Mg^2+^ (*K*_MR2_) could have the same site, too, although it represents the binding to tRNA and not to the E‐tRNA complex. Chloride weakens the binding of Mg^2+^ (*K*_MR2_) and Mg^2+^ (*K*_ME42_).

Mg^2+^ (*K*_MR_) and Mg^2+^ (*K*_ME4_) form another pair, which probably binds to a common site. They cannot be replaced by spermidine, and chloride does not weaken their binding. In the ordered binding to the free tRNA, Mg^2+^ (*K*_MR_) is bound first, and in the ordered dissociation in segment 4 Mg^2+^(*K*_ME4_), it is freed last.

Whether Mg^2+^ (*K*_ME4_) and Mg^2+^ (*K*_ME5_) have the same binding site remains an open question. According to the equations that could be possible, the latter is, however, bound 13 times more strongly. The other possibility of having different sites can also be correct, as removing the term [Mg]/*K*_ME5_ from *C*_54_ leads to only a slightly weaker result in the best‐fit analysis.

In conclusion, in addition to the Mg^2+^ ion in MgATP or MgPP_i_, two or three Mg^2+^ ions participate in the IleRS reaction. One of them can be present in all steps but must be present in the transfer reaction and the reorganizing step before it. The second of them is present only in the transfer reaction and prevents, for example, the activation reaction. A strongly bound Mg^2+^ ion is involved in the events after the transfer reaction.

### Relation to the pretransfer proofreading

During the whole time span of studies on IleRS, understanding the discrimination of the noncognate valine has been one of the key objectives 4. The correction mechanisms include the pretransfer proofreading, where the wrong Val‐AMP is eliminated, and post‐transfer proofreading, where the wrong Val‐tRNA^Ile^ is eliminated. The post‐transfer proofreading has been shown to occur at a separate editing site where the noncognate amino acids are hydrolysed 3.

In recent years, the pretransfer proofreading has received new attention 37, 38, 39. Although it occurs at the aa‐AMP level, it requires tRNA and is believed to be connected to the post‐transfer proofreading site. In the present work, measurements of the proofreading were not taken. However, the existence of both Ile‐AMP and Ile‐tRNA in a ternary complex expands the number of enzyme intermediates that could be involved. If the pretransfer proofreading occurs at segment 6, before the dissociation of Ile‐tRNA from the enzyme, the connection to the post‐transfer editing site could be possible through Ile‐tRNA.

The kinetic proofreading system has been a subject of discussion and mathematical calculations 40, 41, 42. It requires either an increased pyrophosphorolysis rate of the wrong aa‐AMP (in *C*_65_ and *C*_21_) or dissociation of the wrong aa‐AMP from the enzyme and hydrolysis thereafter. These rates can be different in the presence of Ile‐tRNA in segment 6 from that in segment 2. In addition, the Mg^2+^(*K*_ME5_) ion in the presence of Ile‐tRNA can affect the rates.

Still another possibility for the pretransfer proofreading is a hydrolytic elimination of the wrong aa‐AMP on the synthetic site. Even then, the presence of Ile‐tRNA and the Mg^2+^ ion can have their effects.

In the cell, a major part of tRNA is as aa‐tRNA 43. A rough estimate of the strength of binding of Ile‐tRNA to the enzyme can be calculated by analysing the rate curve of the aminoacylation assay by the integrated Michaelis equation 16. This gives the result that the *K*_m_ for tRNA is about twice the value of *K*_p_ for Ile‐tRNA, or Ile‐tRNA is bound more strongly to the enzyme. The high amount of Ile‐tRNA and its stronger binding mean that the amounts of enzyme intermediates with bound Ile‐tRNA in segments 6 and 5 are higher compared to segment 2. This emphasizes segment 6 as the possible site of the pretransfer proofreading.

For the kinetic proofreading, some PP_i_ must be present in *C*_65_ and *C*_21_. Due to the fast pyrophosphorolysis rates (high *k*_‐3_ and *k*_‐35_), the required PP_i_ amount is low, estimated to be below 20 μm. High PP_i_ concentrations (0.5 mm) have repeatedly been measured in *E. coli* cells 44. They cause such strong inhibition that, apparently, high PP_i_ concentrations cannot be in contact with the enzyme. PP_i_ increases the pyrophosphorolysis rates in *C*_21_ and *C*_65_ towards free Ile, and therefore, the advantage of pretransfer proofreading is lost when the corrected Ile from Ile‐AMP is mixed with the original mixture of amino acids.

## Concluding remarks

The key aim of the present work has been to do a best‐fit analysis simultaneously on a large number of different steady‐state kinetic experiments and thus improve the model of the reaction mechanism and the accuracy of the equations. Several improvements could be done, although this kind of kinetic work, naturally, cannot address the mechanism at the molecular level but only at the functional level.

The procedure of the ‘rapid equilibrium segments’ was used in the work. It can be seen that the equations in the procedure rapidly become complicated when the number of the segments increases. The rate equation algorithm contained 25 separate equations. On the other hand, the aminoacyl‐tRNA synthetases are among the most complicated enzymes with three substrates, three products, activators and editing mechanisms; nonetheless, IleRS could be successfully analysed. The principal advantage of the procedure is that only a limited number of equations in the algorithm must be modified when the details of the mechanism in a given segment are tested.
